# A Successful Case of Amaurosis

**Published:** 1809-11

**Authors:** James Gerrard

**Affiliations:** Liverpool


Medical and Phyfical Journal.

VOL. XXII.] NOVEMBER, 1809. [NO. 129.

*Printed for R. PHILLIPS, by W. Thorne, Red Lion Court, Fleet Street, London.*

### *A successful Case of Amaurosis;* by Dr. JAMES GERRARD of Liverpool.

JOHN LAWRENCE, aged 32 years, labouring under amaurosis, admitted into the Liverpool Infirmary the 15th of September, 1791. He is a robust man, a common sailor; has in that capacity experienced all vicissitudes of climate, and has always been accustomed to live freely in every respect.

The disease began in the West Indies, about six months before, without his having been previously exposed to a stronger degree of light than he had frequently before experienced, or any other cause that he could assign.

At this time he was sent to the hospital at Port Royal, Jamaica, where he says various means were used without the least benefit.

He afterwards came to England, and was sent into Haslar Hospital, where, *he says*, the disease was called gutta serena. After remaining there about a month, he was discharged without receiving any relief or encouragement to hope for it. On his admission into the Liverpool Infirmary, he was ordered to use the cold bath, to take the Peruvian bark in powder, and three times a day to instil into the eyes a few drops of an infusion of Cayenne pepper in cold water, in the proportion of one grain to an ounce.

Notwithstanding its having been filtered through paper, it occasioned very considerable pain and a plentiful flow of tears; but the patient being a man of great resolution, he steadily persevered, and was very soon recompensed for his suffering, by the evident advantage which his sight daily received; and though this was but transient in the beginning, yet he was so sensible of it himself, that after having bathed but twice, and taken only a few doses of the bark, he discontinued both, in order to give the infusion a satisfactory trial. Upon finding the effect greater and more lasting on repetition, he used it more frequently and freely than had been directed, or than might have been thought prudent.

As this circumstance seems to have contributed very materially to the success, it appears necessary to describe the particular manner of his using it, which was to lie down on his back, whilst one of the other patients poured the infusion copiously into his eyes; and this was repeated five times a day, although the pain was so great as to occasion an agitation of the whole body. In a few days he was so sensible of the benefit he had derived, that he desired the other patients to hold up different objects betwixt him and the window, to try whether he could tell what they were. On the first attempt he saw them very imperfectly, but he experienced such improvement daily, that he could soon distinguish the church steeples, and other distant objects; and in three weeks from the time of his admission, his sight was so perfectly restored, that he could see as well as ever he had done in his life, and as distinctly as any other patient in the ward. Considering this an uncommonly successful case, I afterwards mentioned it to my colleague, and also to some of the faculty of the dispensary, and regretted that I had not claimed their attention at the time.

An opportunity of correcting that omission, however, occurred last November, when I accidentally met the very man.

I considered this a very fortunate circumstance, as it afforded them the satisfaction not only of seeing him, but also of hearing this account confirmed by himself two years afterward; at the same time he also related some events which had occurred to him since he left the Infirmary, which are perhaps worthy of notice, particularly, that immediately after he was dismissed in October, 1791, he undertook a coasting voyage, in which he was much exposed to the inclemency of the weather, at that season of the year, and was wrecked on the coast of Scotland, in the month of December following; and that besides several other short trips, he had since been a voyage to Africa, where he had continued six months upon the coast Notwithstanding these trials, and his having lived as freely as before, he had experienced the enjoyment of his sight without interruption or abatement during the whole period.

Upon examining his eyes at that time, there was no defect in either, but on the contrary, the iris in each possessed the power of contraction and dilatation in an uncommon uncommon degree, as appeared by the change which evidently and rapidly took place in the pupils, merely on turning his face to the light, or from it; a circumstance which shewed a great degree of sensibility in the retina..

Having now related the case, it is proper to communicate the circumstance which gave rise to the practice, which was mentioned to the Faculty of this Infirmary by the late Mr. Robert Scott, Surgeon of artillery, Woolwich, in justice to whose memory I must acknowledge, that it was on his suggestion that the remedy was first tried here. The occurrence happened in the Bahama Islands, whilst that gentleman was there with the army. Several people were directed to husk some capsicum; amongst them was a blind old man; during his being thus employed, one of his eyes itched; on rubbing it he had excessive pain, owing probably, and as it was supposed, to a particle of pepper getting into his eye; to the man's great surprise, however he could afterward see for a while; the hint was taken, he used an infusion of the pepper, in the manner already described, and he recovered his sight.

